# Prior Therapy With Pegylated-Interferon Alfa-2b Improves the Efficacy of Adjuvant Pembrolizumab in Resectable Advanced Melanoma

**DOI:** 10.3389/fonc.2021.675873

**Published:** 2021-06-16

**Authors:** Dong-Dong Jia, Yanling Niu, Honglin Zhu, Sizhen Wang, Tonghui Ma, Tao Li

**Affiliations:** ^1^ Department of Bone and Soft-tissue Surgery, The Cancer Hospital of the University of Chinese Academy of Sciences (Zhejiang Cancer Hospital), Institute of Basic Medicine and Cancer (IBMC), Chinese Academy of Sciences, Hangzhou, China; ^2^ Department of Translational Medicine, Genetron Health (Beijing) Co., Ltd., Beijing, China

**Keywords:** interferon alpha, adjuvant pembrolizumab, advanced melanoma, sequential therapy, recurrence-free survival

## Abstract

Combination immunotherapy can overcome the limited objective response rates of PD-1 blockade. Interferon alpha (IFN-α) has been proven to be effective in modulating immune responses and may enhance the clinical responses to PD-1 blockade. According to clinical practice guidelines, IFN-α was recommended as adjuvant therapy for stage IIB/C melanoma patients. However, the impact of prior IFN-α therapy on the efficacy of subsequent PD-1 blockade in melanoma has not been previously reported. Therefore, we performed a retrospective analysis for melanoma patients and addressed whether prior IFN-α therapy enhanced adjuvant pembrolizumab as later-line treatment. Fifty-six patients with resectable stage III/IV melanoma who received adjuvant therapy with pembrolizumab were retrospectively enrolled in this study. Notably, 25 patients received adjuvant pegylated IFN-α (PEG-IFN-α) in the prior line of treatment while 31 patients did not receive prior PEG-IFN-α therapy. Cox regression analysis showed that prior PEG-IFN-α therapy was associated with the efficacy of later-line adjuvant pembrolizumab (hazard ratio=0.37, 95% CI 0.16-0.89; *P* = 0.026). The recurrence rates after treatment with adjuvant pembrolizumab were significantly reduced in the prior PEG-IFN-α group (*P* < 0.001). The Kaplan-Meier analysis also showed that recurrence-free survival (RFS) after adjuvant pembrolizumab therapy was prolonged by prior PEG-IFN-α treatment (median RFS_Pem_ 8.5 months *vs*. 4.5 months; *P* = 0.0372). These findings indicated that prior PEG-IFN-α could enhance the efficacy of adjuvant pembrolizumab. The long-lasting effects of PEG-IFN-α provide a new rationale for designing combination or sequential immunotherapy.

## Introduction

Metastatic melanoma is aggressive and has limited treatment options (1). Recently, immune checkpoint blockade with antibodies targeting CTLA-4 and PD-1/PD-L1 has become an important treatment strategy for melanoma patients (2). However, a majority of patients do not respond well to mono-immunotherapy. One strategy to improve efficacy is developing combination therapies (3). Combination of anti-CTLA-4 and anti-PD-1 resulted in higher response rates, although the rates of grade 3/4 adverse events were high (4, 5). Therefore, it is very important to develop other combination/sequential therapies.

Previous studies have demonstrated that CD8+ tumor-infiltrating lymphocytes (TILs) (6) or IFN-γ-related gene expression profiles (7) were predictive of melanoma response to anti-PD-1, suggesting that pre-existing T-cell immune responses may be associated with response to PD-1 blockade. It has also been shown that type I interferons (IFNs), including IFN-α, are capable of enhancing immune responses (8, 9). For instance, type I IFNs stimulate the maturation of dendritic cells, promote the survival of CD8+ T cells, enhance the release of pro-inflammatory cytokines by macrophages, increase PD-L1 expression on immune cells, and inactivate the suppressive functions of regulatory T cells (10–16). IFN-α in the adjuvant setting was approved for patients with stage IIB/C melanoma and was very likely used as a frontline treatment in clinics. The results from the phase Ib/II KEYNOTE-020 trial showed that pembrolizumab/PEG-IFN-α combination resulted in promising clinical efficacy in advanced melanoma (9). Considering the functions of IFN-α in immune responses and its clinical use in stage IIB/C melanoma, we tried to investigate whether a prior line of PEG-IFN-α therapy would affect the efficacy of later adjuvant pembrolizumab.

In the present study, we retrospectively assessed patients with resectable stage III/V melanoma treated with adjuvant pembrolizumab therapy. We demonstrated for the first time that prior PEG-IFN-α therapy enhanced the efficacy of later adjuvant pembrolizumab. These data suggested that administration of PEG-IFN-α had a relatively long-lasting effect on the adjuvant pembrolizumab response.

## Methods

### Study Participants and Treatment

**Figure 1:**
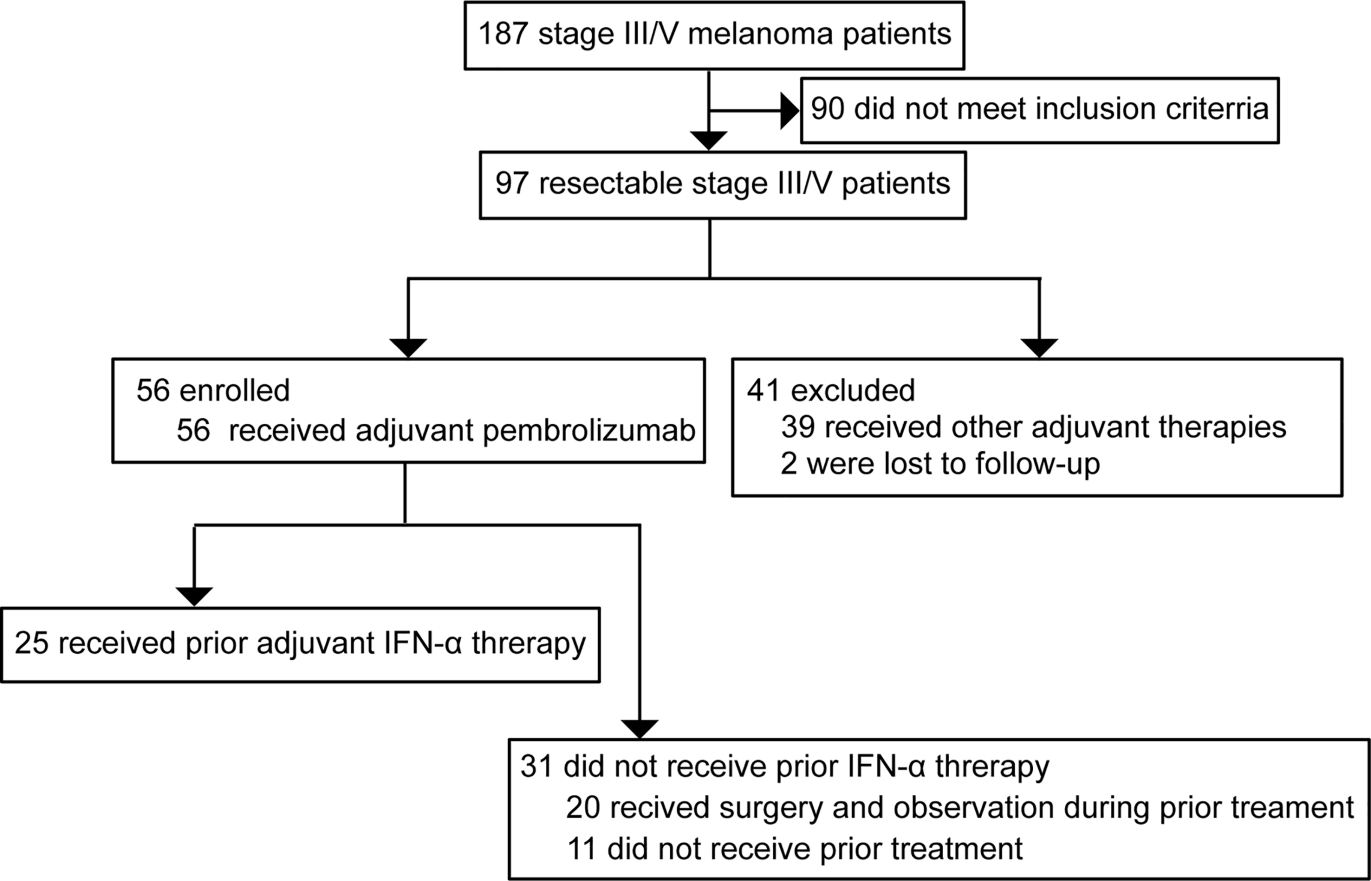
Study design, screening, and enrollment of the participants.

All melanoma patients treated at the Cancer Hospital of the University of Chinese Academy of Sciences (Zhejiang Cancer Hospital) between 2018 and 2020 were screened and 97 patients with stage III (with nodal or local satellite/in-transit metastases) or oligometastatic stage IV resectable melanoma were selected. Of those, 56 patients treated with adjuvant pembrolizumab were enrolled. Among them, 25 patients had received adjuvant pegylated-interferon alfa-2b (PEG-IFN-α) as the prior line of treatment. For those without prior PEG-IFN-α therapy, 20 patients had received surgery of primary lesions followed by observation during prior treatment and 11 patients had never received prior therapy. The patients treated with other prior therapies were excluded from the analysis (**Figure 1**). The treatment timeline for all patients is shown in **Figure S1**. High-dose adjuvant PEG-IFN-α therapy was administered for these 25 patients. All the enrolled patients were intravenously given adjuvant pembrolizumab therapy every 3 weeks for up to 18 doses. Regular physical examinations were performed during treatment. All participants were pathologically confirmed acral (n=40) or cutaneous (n=16) melanomas. The demographic and clinical characteristics of all study participants are presented in **Table 1**. This study was approved by the ethics committee of Zhejiang Cancer Hospital. All patients provided written informed consent.

**Table 1:** Demographic and clinical characteristics of patients.

Patient characteristics	IFN-α (n=25)	no IFN-α (n=31)	P value
**Sex**			
Female	16 (64%)	19 (61%)	0.835
Male	9 (36%)	12 (39%)	
**Age (years)**			
<60	13 (52%)	15 (48%)	0.788
≥60	12 (48%)	16 (52%)	
**Stage**			
III	17 (68%)	24 (77%)	0.429
IV	8 (32%)	7 (23%)	
**Site of primary tumor**			
Acral	19 (76%)	21 (68%)	0.496
Cutaneous	6 (24%)	10 (32%)	
**Ulceration**			
Yes	7 (28%)	13 (42%)	0.239
No	18 (72%)	17 (55%)	
Unknown/Missing	0 (0%)	1 (3%)	
**Breslow Thickness**			
≤4mm	8 (32%)	14 (45%)	0.137
>4mm	11 (44%)	6 (19%)	
Unknown/Missing	6 (24%)	11 (35%)	
**No of metastases**			
≤3	13 (52%)	19 (61%)	0.396
>3	12 (48%)	11 (35%)	
Unknown/Missing	0 (0%)	1 (3%)	
**NRAS mutation status**			
Wildtype	21 (84%)	24 (77%)	0.781
Mutation	4 (16%)	7 (23%)	
**BRAF mutation status**			
Wildtype	21 (84%)	25 (81%)	1.000
Mutation	4 (16%)	6 (19%)	
**CCND1 amplification status**			
Yes	3 (12%)	3 (10%)	1.000
No	22 (88%)	28 (90%)	
**PD-L1 expression (≥upper quartile *vs*. <upper quartile)**			
≥15%	6 (24%)	8 (26%)	0.247
<15%	15 (60%)	22 (71%)	
Unknown/Missing	4 (16%)	1 (3%)	
**TMB (≥upper quartile *vs*. <upper quartile)**			
≥10 mut/Mb	7 (28%)	6 (19%)	0.446
<10 mut/Mb	18 (72%)	25 (81%)	

There were no significant between-group differences in the characteristics listed here. Gene mutations or TMB values were detected or calculated by NGS targeting a panel of cancer-related genes. PD-L1 expression in tumor and tumor-associated immune cells was assessed using a 22C3 antibody assay.

### Targeted Next-Generation Sequencing and PD-L1 Testing

Targeted next-generation sequencing (NGS) was performed on the matched tumor and blood specimens (Onco PanScan™). The sequencing data were analyzed to detect genomic alterations, copy number variations, and tumor mutation burden (TMB). Membranous expression of PD-L1 was assessed by the immunohistochemistry assay (22C3 antibody).

### Statistical Analysis

The Kaplan-Meier method was used for survival analysis and statistical significance was assessed using the Log-rank test method. RFS_Pem_ was defined as the time between the date of starting adjuvant pembrolizumab and the date of recurrence after administering adjuvant pembrolizumab. The association of variables with RFS_Pem_ was determined by the Cox proportional-hazards model. Hazard ratios and 95% confidence intervals were calculated from the Cox regression model. The recurrence rates after administering adjuvant pembrolizumab during different time-points were compared between the IFN-α and no IFN-α groups using the Chi-Square Test. Statistical significance was set at 0.05. All statistical analyses were performed using SPSS version 25.0.

## Results

### Treatment Strategies and Clinicopathological Characteristics for Patients

A total of 56 patients with stage III/IV resectable melanoma treated with adjuvant pembrolizumab therapy were retrospectively assessed in this study. Treatment process for all the patients was shown in **Figure S1**. Based on medical history, 25 patients received PEG-IFN-α as the prior line of adjuvant treatment (IFN-α group) while 31 patients who received surgical resection of primary lesions or did not receive prior treatment were assigned to the control group (no IFN-α group). The selection of adjuvant PEG-IFN-α treatment was based on the stage of disease and patients’ willingness. Recurrence-free survival after adjuvant pembrolizumab (RFS_Pem_) treatment was explored. The median follow-up time for the IFN-α group was 12.3 months and the median follow-up duration for patients without IFN-α treatment was 11.3 months. Clinicopathological characteristics are listed in **Table 1**. About 71% patients (n=40) were acral melanoma and 29% patients (n=16) were cutaneous melanoma. Demographic characteristics of these patients showed no difference between the IFN-α and no IFN-α groups.

### The Genomic Alterations of Patients

**Figure 2:**
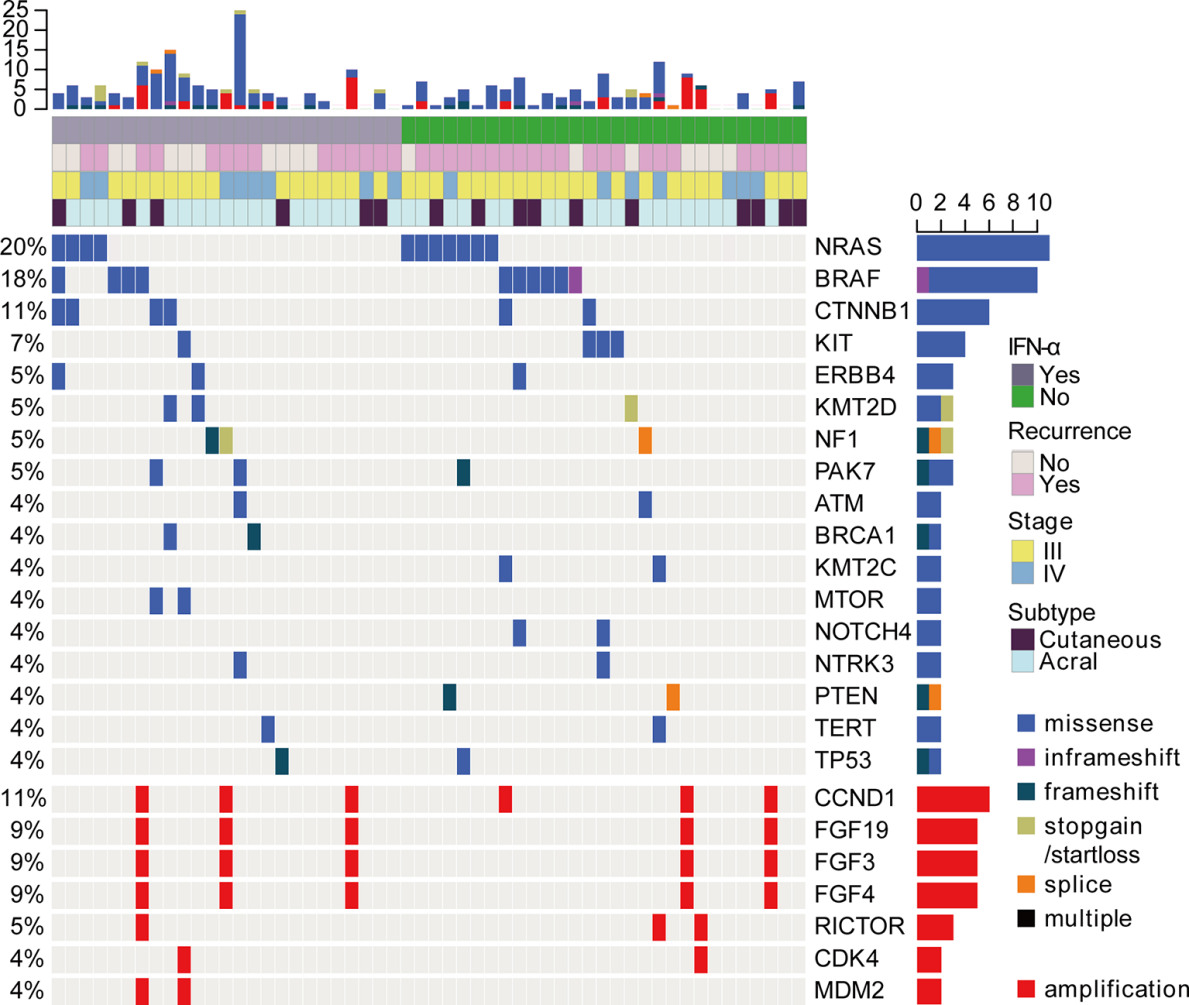
The landscape of driver mutations and CNVs in acral (n = 40) and cutaneous (n = 16) melanomas by targeted NGS. Each column represents a case and each row represents a gene. The very top bar graph shows the number of mutations and CNVs detected in each sample. Recurrence after adjuvant pembrolizumab treatment, prior treatment, stage, and subtype are represented in the rows. The number on the left indicates the percentage of mutations or CNVs in 56 samples. The bar graph on the right shows the total number of mutations or CNVs for each gene. Mutation profiling was shown in the top panel. Copy number analysis was also analyzed and shown in the bottom panel.

We examined somatic exonic mutations in a subset of cancer-related genes in the tumors (**Figure 2**). The results revealed that *NRAS* gene was the most frequently mutated gene (20%, 11 of 56). While *BRAF* mutations accounted for 18% (10 of 56) of mutations. The previous data showed that about 52% of patients harbored *BRAF* somatic mutations in cutaneous melanoma and the proportion of *BRAF* mutations was relatively lower in acral melanoma (17, 18). The frequency of *BRAF* mutations was even lower in this study than previously reported. This may be because of the higher percentage of acral melanomas in our cohort. It is also possible that some *BRAF* V600E/K mutant patients received BRAF inhibitors as adjuvant therapy and were therefore not included. Additionally, *CTNNB1* and *KIT* mutations were present in 11% (6 of 56) and 7% (4 of 56) of the participants respectively. Analysis of copy number variations (CNVs) revealed that the *CCND1*/*FGF3*/*FGF4*/*FGF19* amplifications were the most common CNVs in this cohort. Importantly, no significant differences in the distribution of genomic alterations, such as *BRAF* mutations, *NRAS* mutations, and *CCND1* amplification, could be found between the IFN-α and no IFN-α groups (**Table 1** and **Figure 2**).

The amplified genes (*MDM2*/*MDM4*/*CCND1*/*FGF3*/*FGF4*/*FGF19*) have been suggested as genomic correlates of increased risk of hyperprogression after immune checkpoint inhibitors (19). Another study reported that *CDK4* copy number gain was negatively correlated with anti-PD1 response in all subtypes of advanced melanoma and *CCND1* copy number gain was associated with a lack of response to anti-PD-1 therapy in advanced acral melanoma (20). However, there were no reports to study the associations between CNVs and clinical benefits of adjuvant anti-PD-1 immunotherapy in melanoma. Then we divided the patients into two groups according to RFS_Pem_ (RFS_Pem_ ≤ 4 months *vs*. RFS_Pem_ > 4 months). The data revealed that the proportions of patients with *CCND1* amplification or *FGF3*/*FGF4*/*FGF19* co-amplification were higher in the rapid progression group (RFS_Pem_ ≤ 4 months), but the difference was not statistically significant (**Figure S2**). Here, amplifications of these genes did not reveal a significant association with clinical responses to adjuvant pembrolizumab.

### Subgroup Analysis of the Efficacy of Adjuvant Pembrolizumab

**Figure 3:**
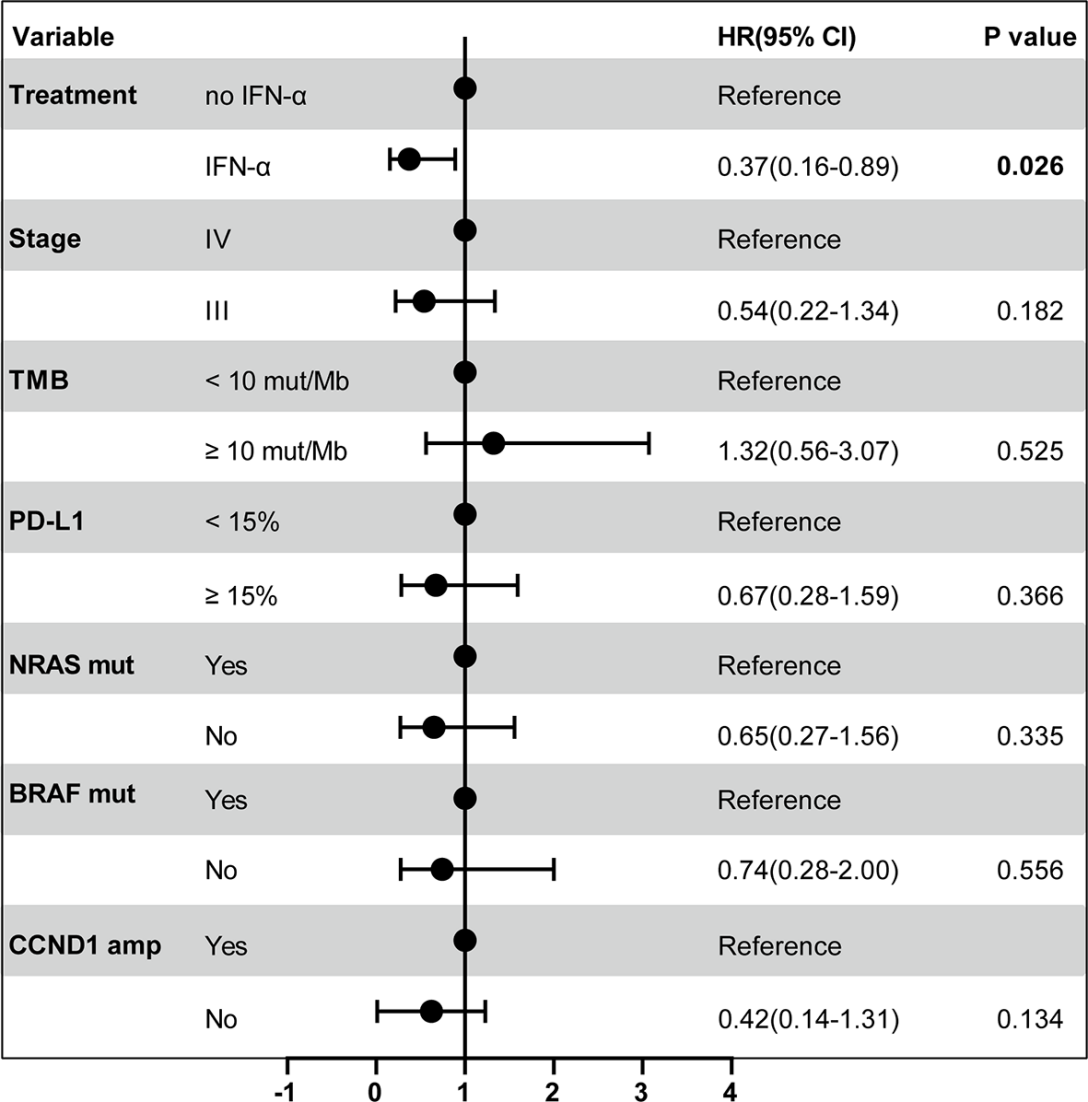
Forest plot of RFS_Pem_ according to the prespecified subgroups. The multivariable Cox proportional-hazards model was used to estimate the hazard ratios for the risk of recurrence after adjuvant pembrolizumab among all patients. The solid circle indicates hazard ratio (HR) for the risk of recurrence. The bar represents the 95% confidence interval (CI) for each HR. The P value for the comparison is also represented.

High levels of TMB and PD-L1 expression are considered to be the predictive biomarkers for PD-1 blockade in many cancer therapies. For example, TMB-high was associated with a survival benefit for metastasized or unresectable melanoma patients with ipilimumab (anti-CTLA-4) and nivolumab (anti-PD-1) therapy (21). Melanoma patients with positive PD-L1 expression and smaller tumors had a higher complete response with pembrolizumab treatment (22). In addition, IFN-α might enhance clinical benefits from PD-1 blockade by stimulating immune responses. To investigate the associations between different variables with RFS_Pem_ and identify patients who may benefit from adjuvant pembrolizumab, we generated a Cox proportional-hazards model. As shown in **Figure 3**, demographic characteristic (stage), molecular biomarkers (TMB and PD-L1), frequently mutated genes (*BRAF* and *NRAS*), amplification of *CCND1* gene did not have any associations with the differences in RFS_Pem_. However, prior clinical treatment (IFN-α) was significantly associated with the efficacy of adjuvant pembrolizumab (hazard ratio (HR)=0.37, 95% CI 0.16-0.89; *P* = 0.026).

### Prior PEG-IFN-α Therapy Enhances the Efficacy of Adjuvant Pembrolizumab

**Figure 4:**
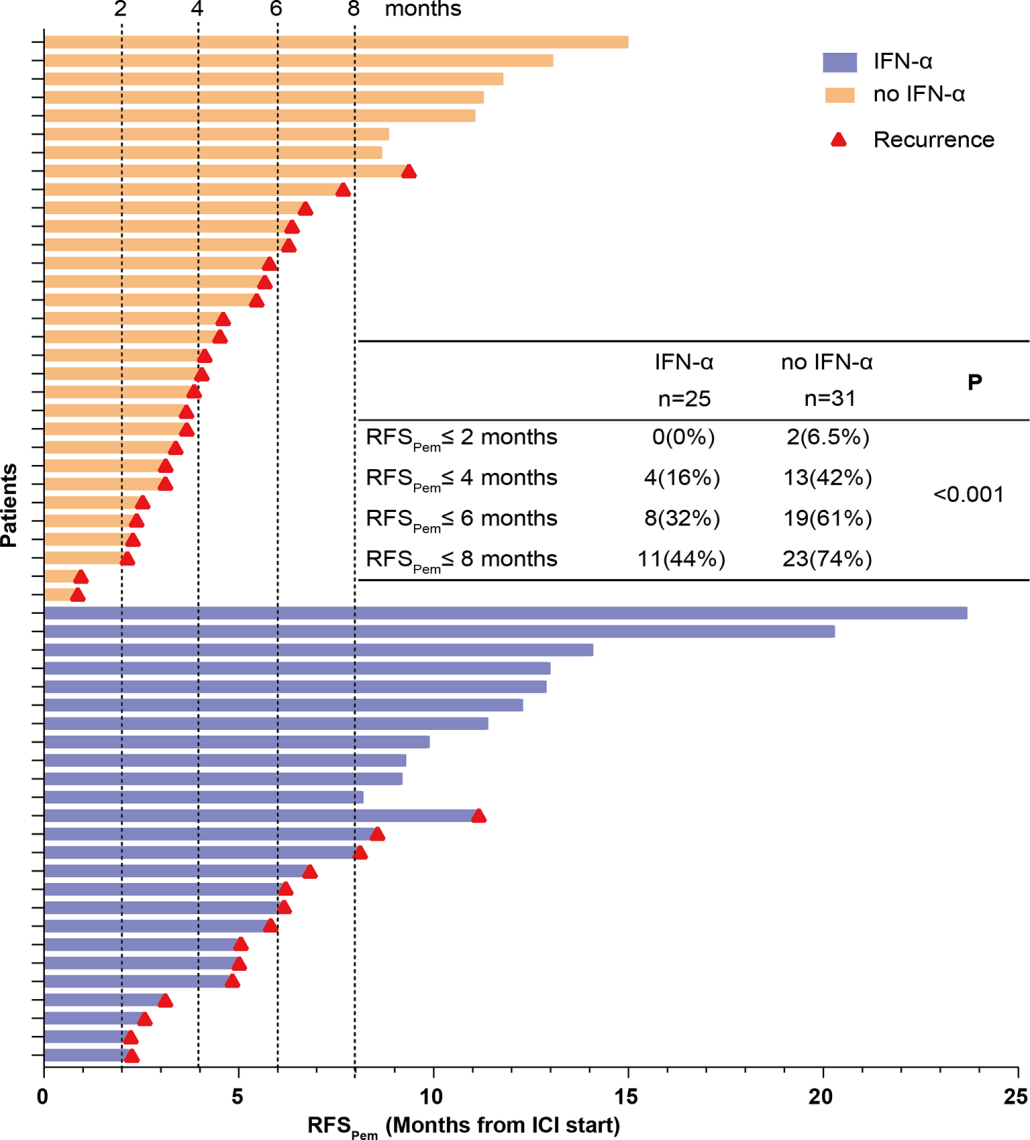
RFS_Pem_ of all patients was summarized. Each row represents one patient. The length of each bar shows the time from start of adjuvant pembrolizumab treatment to the recurrence or the last follow-up. The red triangle indicates patients who developed a recurrence. The numbers and proportions of patients who developed a recurrence at different time points were calculated. The P value was generated from the Chi-Square test to compare the recurrence rates between the IFN-α and no IFN-α groups.

According to the results of Cox proportional-hazards model, we further investigated whether prior PEG-IFN-α treatment enhanced clinical benefits of adjuvant pembrolizumab. The effects of prior PEG-IFN-α on the efficacy of pembrolizumab were shown in **Figure 4**. In the control group (no IFN-α, orange columns), 6.5% (2 of 31) and 42% (13 of 31) of the patients had a recurrence within 2 and 4 months, respectively. In contrast, no patients had a relapse within 2 months and only 16% (4 of 25) developed a recurrence within 4 months in the IFN-α group (blue columns). The recurrence rates were significantly lower in the IFN-α group than in the no IFN-α group within 6 or 8 months. This suggested that prior PEG- IFN-α treatment enhanced the clinical effects of adjuvant pembrolizumab dramatically (*P* < 0.001).

**Figure 5:**
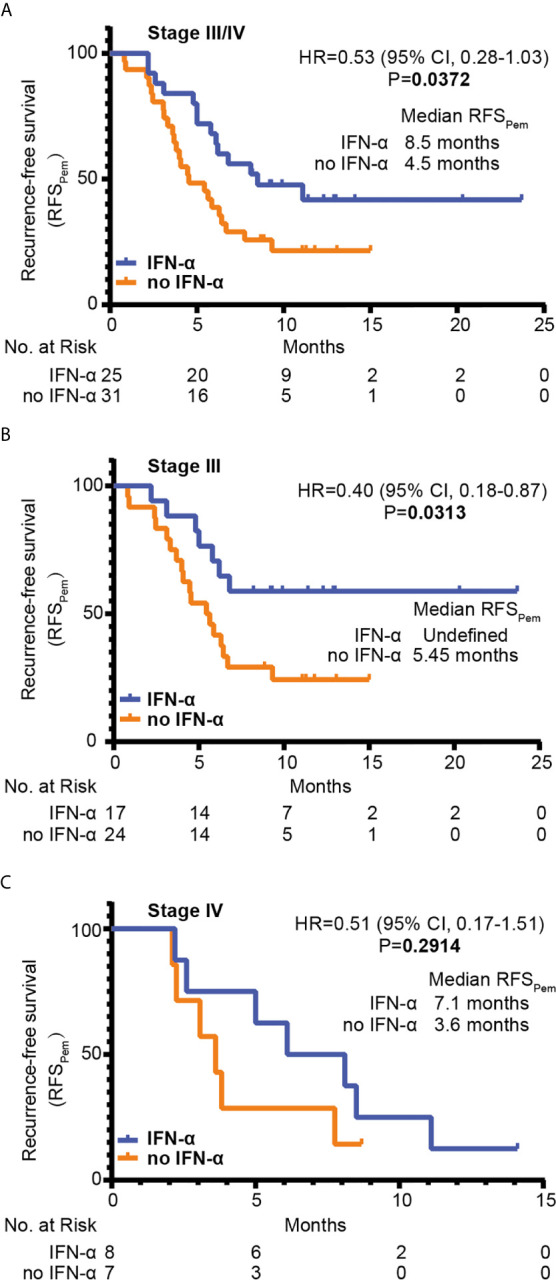
The RFS_Pem_ of patients was estimated with the Kaplan-Meier method. The numbers of patients at risk at each time point were shown below. Statistical analysis was performed by the Log-rank test between two groups. RFS_Pem_ of all enrolled patients was shown in **(A)** RFS_Pem_ of patients with stage III melanoma (n=40) was shown in **(B)** RFS_Pem_ of patients with stage IV melanoma (n=16) was shown in **(C)** HR, hazard ratio; CI, confidence interval.

The RFS_Pem_ distribution was also estimated with the Kaplan-Meier method, as shown in **Figure 5**. RFS_Pem_ was significantly prolonged in the IFN-α group compared to the no IFN-α group (median RFS_Pem_ 8.5 months *vs*. 4.5 months, HR=0.53, 95% CI 0.28-1.03; *P* = 0.0372) (**Figure 5A**). In stage III melanoma, the median RFS_Pem_ for the IFN-α group was not reached while it was 5.45 months in the no IFN-α group (HR=0.40, 95% CI 0.18-0.87; *P* = 0.0313) (**Figure 5B**). For patients with stage IV melanoma, there was a trend which suggested that median RFS_Pem_ in the IFN-α group was higher compared with the no IFN-α group. However, this trend did not reach statistical significance (median RFS_Pem_ 7.1 months *vs*. 3.6 months, HR=0.51, 95% CI 0.17-1.51; *P* = 0.2914) (**Figure 5C**). Subgroup analysis on the impact of prior PEG-IFN-α treatment on RFS_Pem_ showed a trend towards better clinical response to adjuvant pembrolizumab for patients with acral melanoma who received prior PEG-IFN-α therapy (median RFS_Pem_ 8.5 months *vs*. 5.4 months, **Figure S3A**). We also found the RFS_Pem_ benefit for patients with cutaneous melanoma who were pretreated with IFN-α (median RFS_Pem_ 14.4 months *vs*. 3.2 months, **Figure S3B**), but the difference was not significant. Based on the data shown above, we could conclude that prior treatment with PEG-IFN-α significantly enhanced the efficacy of later adjuvant pembrolizumab.

## Discussion

In this retrospective study, we for the first time demonstrated that the prior line of PEG-IFN-α treatment dramatically promoted the efficacy of later-line adjuvant pembrolizumab. Therefore, PEG-IFN-α had a long-lasting effect on sensitizing later-line pembrolizumab treatment by reducing the risk of recurrence. There are some possible explanations for the effect of PEG-IFN-α on immunotherapy. First, IFN-α exerts its effect on immune cells and may preset the immune system to condition which is favorable for pembrolizumab. IFN-α directly activates immune cells, including CD8+ T, dendritic, and natural killer cells (8, 12, 16, 23, 24). IFN-α can also disarm regulatory T cells (15). Second, IFN-α up-regulates the expression of PD-L1 (13), which is the most studied predictive biomarker for response to immune checkpoint blockade. Third, IFN-γ-related mRNA profile predicts response to PD-1 blockade (7). Regulation on transcription profiles by IFN-α may also be associated with the efficacy of immunotherapy. Therefore, further investigation is required to dissect how IFN-α enhances the efficacy of PD-1 blockade. To answer this question, animal studies can be performed to address the effect of IFN-α on immune cells, tumor-infiltrating lymphocytes, transcriptional profiles, and predictive biomarkers of anti-PD-1 therapy.

The phase Ib/II study revealed that pembrolizumab/PEG-IFN-α combination resulted in very promising clinical efficacy in PD-1-naϊve advanced melanoma (9). By contrast, the treatment process for patients in our study was sequential therapy rather than combination therapy. Both studies support new rationale for investigating the clinical efficacy of IFN-α/other PD-1 inhibitors combination or sequential therapies in melanoma. However, the sample size in our study is not big and a large-cohort prospective study is needed to evaluate the efficacy of IFN-α/pembrolizumab sequential therapy.

The median RFS_Pem_ in this study was shorter compared to the KEYNOTE-054 trial, in which the patients had either stage IIIA, or IIIB or IIIC melanoma with no in-transit metastases (25). By contrast, we analyzed stage III melanoma (with local or satellite/in-transit metastases) or oligometastatic stage IV melanoma. These data indicated that patients with more advanced melanoma may have higher risk of relapse with adjuvant pembrolizumab. Moreover, the median RFS in the trial of adjuvant nivolumab versus ipilimumab for resected stage IIIB, IIIC, or IV melanoma patients (CheckMate 238) was also longer than RFS_Pem_ in our analysis. Only a small fraction of patients was acral melanoma in the CheckMate 238 trial, which might account for the difference in RFS, as presumably anti-PD-1 therapy was less effective against acral melanoma compared with cutaneous melanoma (26–28). In addition, most patients had already developed a recurrence after prior treatment for stage IIB/C disease and they might be at a much higher risk of relapse on adjuvant pembrolizumab than patients without prior relapse.

Many clinical trials have been done to investigate adjuvant treatment with IFN-α for stage IIB/C melanoma patients, but the results varied across different trials (29, 30). A meta-analysis of fifteen randomized trials revealed small, statistically significant improvements in both RFS and OS (31). Therefore, adjuvant IFN-α is no longer proposed routinely for cutaneous melanoma by the recently published NCCN guidelines (32). However, the effect of adjuvant IFN-α in acral melanoma needs further investigation. Then we determined whether adjuvant PEG-IFN-α was effective on recurrence-free survival (RFS_1_) in our cohort. The Kaplan-Meier analysis showed that adjuvant PEG-IFN-α could prolong RFS_1_ and delay tumor progressing into advanced stages (*P* = 0.0716, **Figure S4**). The meta-analysis also demonstrated that patients with ulcerated tumors might obtain higher benefit form adjuvant IFN-α (31). In our study, ulceration of the primary tumors was not a criterion for adjuvant treatment of IFN-α. Because the enrolled patients received adjuvant IFN-α ahead of that report. In addition, we did not find that patients with ulcerated melanoma had a better clinical response to adjuvant pembrolizumab (data not shown).

In summary, our data showed that adjuvant PEG-IFN-α had some effects on reducing the risk of recurrence after its clinical use for stage IIB/C melanoma. More importantly, prior PEG-IFN-α treatment could enhance the efficacy of adjuvant pembrolizumab in resected advanced melanoma, suggesting the importance of IFN-α in clinical applications. All these findings suggested that sequential therapy with IFN-α and PD-1 blockade may have great potential for treating melanoma patients.

## Data Availability Statement

The original contributions presented in the study are publicly available. This data can be found here: https://www.biosino.org/node/, accession number OEP002322.

## Ethics Statement

The studies involving human participants were reviewed and approved by the medical ethics committee of Zhejiang Cancer Hospital. The patients/participants provided their written informed consent to participate in this study. Written informed consent was obtained from the individual(s) for the publication of any potentially identifiable images or data included in this article.

## Author Contributions

D-DJ, YN, and TL had full access to all of the data in the study and took responsibility for the integrity of the data and the accuracy of the data analysis. Conception/Design: D-DJ, YN, TM, and TL. Provision of study material or patients: D-DJ and TL. Collection and/or assembly of data: All authors. Data analysis and interpretation: D-DJ and YN. Manuscript writing: D-DJ and YN. Study supervision: TL. All authors contributed to the article and approved the submitted version.

## Conflict of Interest

Authors TM, YN, HZ, and SW were employed by the company Genetron Health (Beijing) Co., Ltd.

The remaining authors declare that the research was conducted in the absence of any commercial or financial relationships that could be construed as a potential conflict of interest.

## Abbreviations

IFN-α, Interferon alfa; PEG-IFN-α, Pegylated-interferon alfa-2b; RFS, Recurrence-free survival; OS, Overall survival; NGS, Next-generation sequencing; CNVs, copy number variations.
