# Supplementary material for: Prior Therapy With Pegylated-Interferon Alfa-2b Improves the Efficacy of Adjuvant Pembrolizumab in Resectable Advanced Melanoma
Source: Front Oncol. 2021 Jun 16;11:675873. doi: 10.3389/fonc.2021.675873 (PMC8243982; doi:10.3389/fonc.2021.675873)

**Supplementary figure S1** The treatment timeline for all patients. The arrow indicates the time of surgery for primary lesions in 56 patients. The triangle indicates the time of surgery for recurrent/metastatic lesions. The circle represents the time of starting adjuvant IFN-α. The rectangle represents the time of starting adjuvant pembrolizumab.
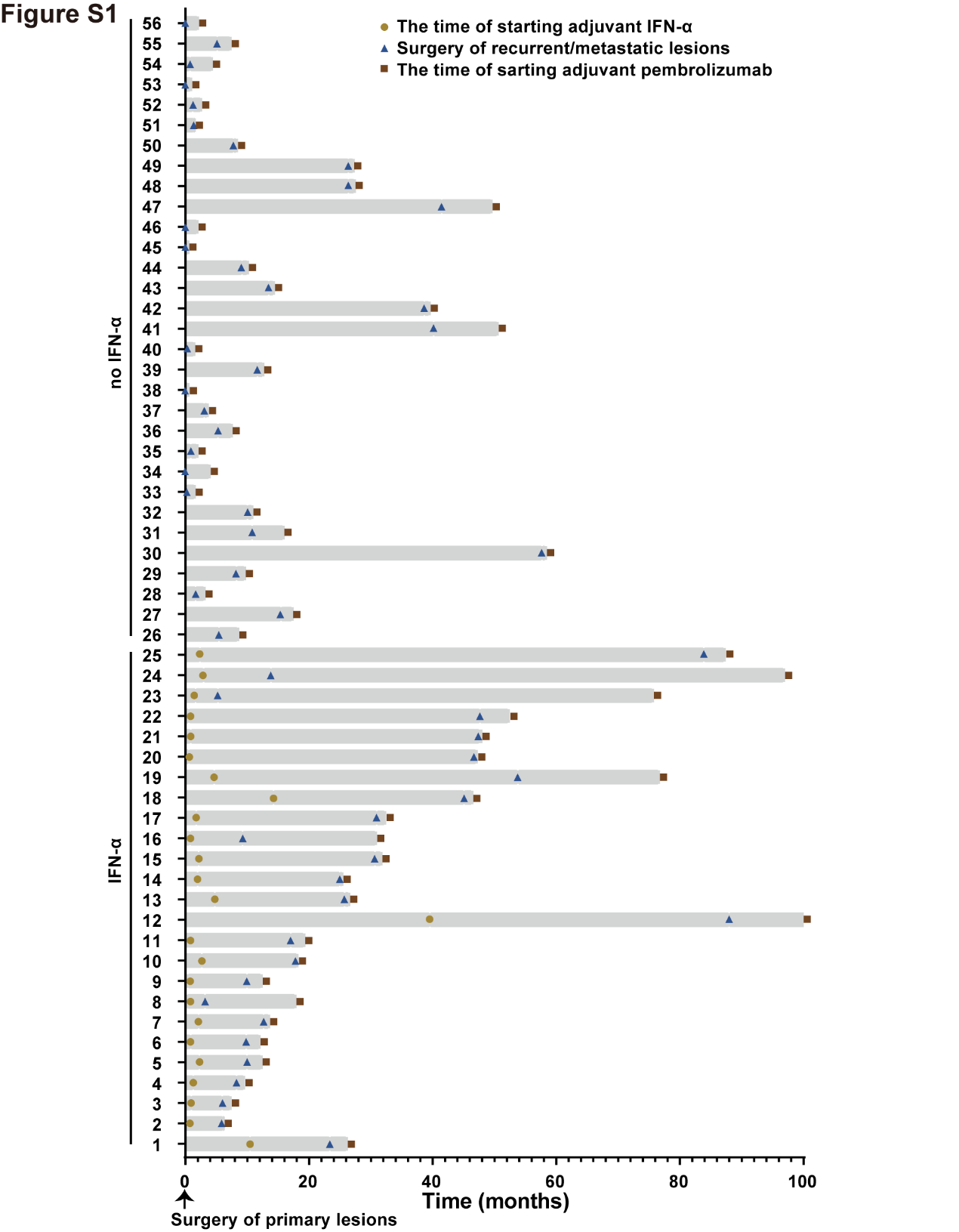


**Supplementary figure S2** Effects of copy number variations (CNVs) on response to adjuvant pembrolizumab in melanoma. Patients were grouped by RFS_Pem_ (RFS_Pem_ ≤ 4 months and RFS_Pem_ > 4 months). The proportions of patients who had gene amplifications were calculated.


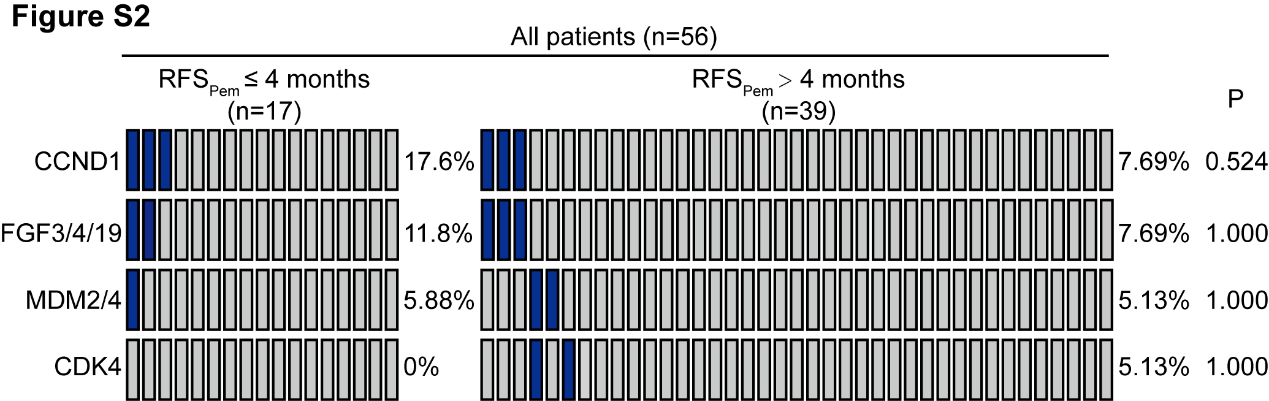


**Supplementary figure S3** The RFS_Pem_ of patients was estimated in acral and cutaneous melanoma. RFS_Pem_ of patients with acral melanoma is shown in **A**. RFS_Pem_ of patients with cutaneous melanoma is shown in **B**.


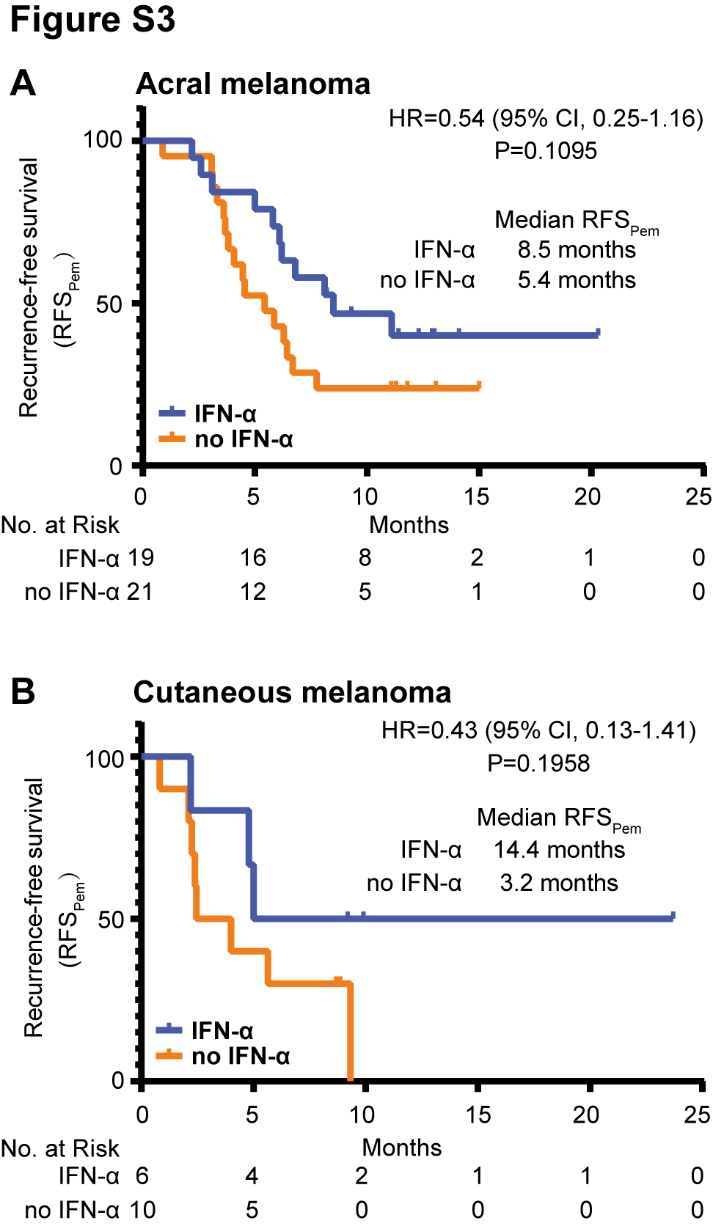


**Supplementary figure S4** The RFS_1_ of patients was represented and estimated with the Kaplan-Meier method. The numbers of patients at risk at each time point were shown below. Statistical analysis was performed by the Log-rank test between two groups. RFS_1_ was defined as the time between the date of first surgery for primary lesions and the date of first recurrence.


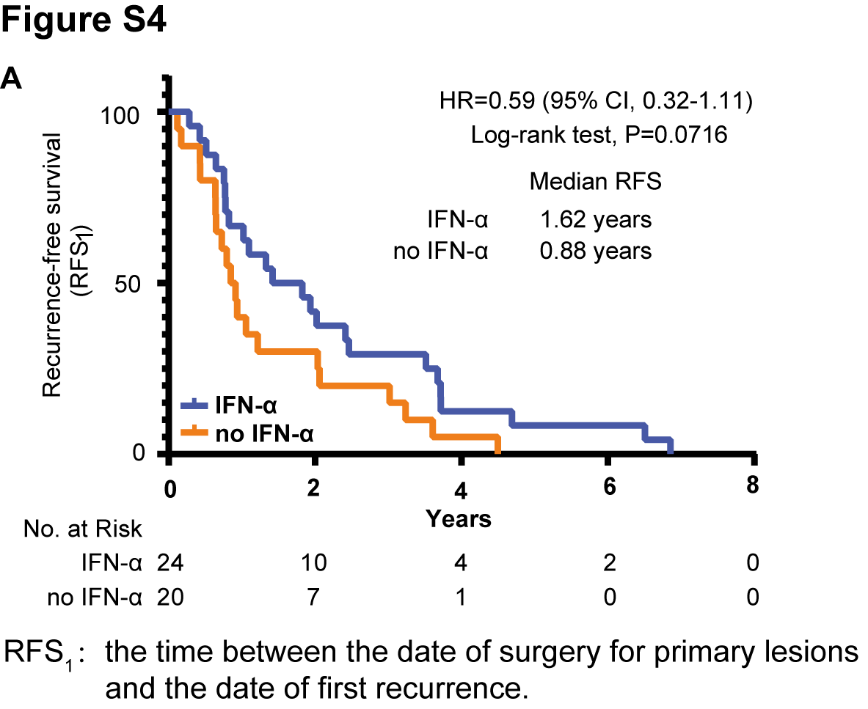

Supplement: Supplementary file 1 [file DataSheet_1.docx]
